# Adsorption of Crystal Violet Dye Using Activated Carbon of Lemon Wood and Activated Carbon/Fe_3_O_4_ Magnetic Nanocomposite from Aqueous Solutions: A Kinetic, Equilibrium and Thermodynamic Study

**DOI:** 10.3390/molecules26082241

**Published:** 2021-04-13

**Authors:** Rauf Foroutan, Seyed Jamaleddin Peighambardoust, Seyed Hadi Peighambardoust, Mirian Pateiro, Jose M. Lorenzo

**Affiliations:** 1 Faculty of Chemical and Petroleum Engineering, University of Tabriz, Tabriz 5166616471, Iran; r.foroutan@tabrizu.ac.ir (R.F.); j.peighambardoust@tabrizu.ac.ir (S.J.P.); 2 Department of Food Science, College of Agriculture, University of Tabriz, Tabriz 5166616471, Iran; peighambardoust@tabrizu.ac.ir; 3 Centro Tecnológico de la Carne de Galicia, Rúa Galicia No. 4, Parque Tecnológico de Galicia, 32900 San Cibrao das Viñas, Ourense, Spain; mirianpateiro@ceteca.net; 4 Área de Tecnología de los Alimentos, Facultad de Ciencias de Ourense, Universidad de Vigo, 32004 Vigo, Ourense, Spain

**Keywords:** activated carbon, magnetic nanoparticles, aqueous solution, cationic dye, isothermal models

## Abstract

Activated carbon prepared from lemon (*Citrus limon*) wood (ACL) and ACL/Fe_3_O_4_ magnetic nanocomposite were effectively used to remove the cationic dye of crystal violet (CV) from aqueous solutions. The results showed that Fe_3_O_4_ nanoparticles were successfully placed in the structure of ACL and the produced nanocomposites showed superior magnetic properties. It was found that pH was the most effective parameter in the CV dye adsorption and pH of 9 gave the maximum adsorption efficiency of 93.5% and 98.3% for ACL and ACL/Fe_3_O_4_, respectively. The Dubinin–Radushkevich (D-R) and Langmuir models were selected to investigate the CV dye adsorption equilibrium behavior for ACL and ACL/Fe_3_O_4_, respectively. A maximum adsorption capacity of 23.6 and 35.3 mg/g was obtained for ACL and ACL/Fe_3_O_4_, respectively indicating superior adsorption capacity of Fe_3_O_4_ nanoparticles. The kinetic data of the adsorption process followed the pseudo-second order (PSO) kinetic model, indicating that chemical mechanisms may have an effect on the CV dye adsorption. The negative values obtained for Gibb’s free energy parameter (−20 < ΔG < 0 kJ/mol) showed that the adsorption process using both types of the adsorbents was physical. Moreover, the CV dye adsorption enthalpy (ΔH) values of −45.4 for ACL and −56.9 kJ/mol for ACL/Fe_3_O_4_ were obtained indicating that the adsorption process was exothermic. Overall, ACL and ACL/Fe_3_O_4_ magnetic nanocomposites provide a novel and effective type of adsorbents to remove CV dye from the aqueous solutions.

## 1. Introduction

Water is one of the most important and effective substances in human life and other living beings. It is used every day in various industries and is exposed to various chemicals and thus can be contaminated. In recent decades, water pollution has become a serious threat to the environmental system, therefore, reducing pollutants from industrial wastewater before discharging to the environment is necessary [1]. The industrial wastewater usually contains several organic and toxic substances that can be harmful to human and aquatic life [2]. Dyes are the first known contaminants in the industrial wastewater streams. Various industries such as food processing, paper, cosmetics, leather, textiles, printing and pharmaceuticals discharge large amounts of wastewater containing dyes polluted with toxic compounds into the environment [3,4]. Annually, it is estimated that 50,000 tons of organic dyes are disposed of worldwide [5].

Dyes used in industry are classified into three categories of cationic dyes (all base dyes), anionic (direct, acidic and reactive dyes) and non-ionic (disperse dyes) [6,7]. Cationic dyes, being as more dangerous than other types, are widely used in the industry. It is reported that 12% of the annual production (about 700,000 tons) of cationic dyes is wasted through industrial water streams polluting the environment [8,9]. Crystal violet (CV) dye is a cationic dye of triphenyl methane with high intensity and it is used in various industries such as pharmaceuticals, paper, textiles and printing inks [10]. CV dye is more toxic than negative dye [11] and if present in water can reduce the penetration of sunlight and disrupt the process of photosynthesis [12]. In addition, CV dye in certain concentrations can cause various diseases and illnesses such as respiratory failure, eye irritation, increased heart rate, skin irritation, blindness, cyanosis, cancer and mutagenesis [10]. Therefore, it is necessary to remove the CV dye from industrial wastewater streams before entering the environment.

Numerous methods have been used to remove various dyes from aqueous solutions and industrial wastewaters, among which nanofiltration, ozonation, flocculation, reverse osmosis, adsorption, electrochemical and biological degradation, chemical oxidation, and photocatalytic degradation are the most extensively used methods [13,14]. Despite to establishing such methods, attempts to find suitable methods with high efficiency, low cost and ease of process are scarce [11]. Adsorption is one of the methods received a lot of attention due to its advantages such as being cheap, having process flexibility with no sludge production, the process simplicity, efficiency and high speed [15,16]. Conventional adsorbents such as activated carbon, biowaste and clay have been used to remove dyes from water, but most of them have low adsorption capacity with low selectivity [17]. In this regard, the use of nanoparticles (magnetic or non-magnetic) to produce adsorbents with high active surface is a very effective method, because nanoparticles owing to their porous structure and high surface area can improve the efficiency of the adsorption process in removing dye substances [18]. The advantage of using magnetic nanoparticles over non-magnetic ones in addition to improving the adsorbent efficiency, is that they can be easily separated from aqueous solutions [19]. Fe_3_O_4_ magnetic nanoparticles provide an additional advantage of being economic with low toxicity can be used in the adsorption process for the removal of various pollutants [20].

In the adsorption process, various adsorbents such as activated carbon, natural fibers, carbon nanotubes, zeolites, polymeric materials [21] and magnetic nanocomposite [4,22] have been used. However, there is a room for new challenges in the development of novel materials as adsorbents in the water treatment process. In the present study, lemon wood was used as a suitable primary source in the preparation of activated carbon and activated carbon/Fe_3_O_4_ magnetic nanocomposite and the ability and efficiency of these nanocomposites in removing CV dye from aqueous solution were evaluated. In addition, the effects of parameters such as temperature, contact time, adsorbent dose, initial CV dye concentration and contact time on the CV dye adsorption process were investigated.

## 2. Results and Discussion

### 2.1. Characteristics of Nanocomposites

**Figure 1:**
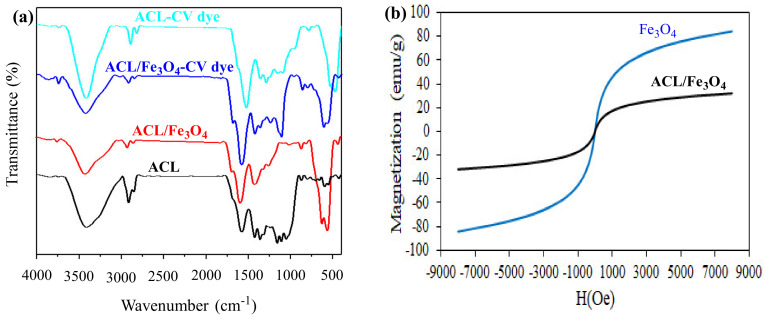
(**a**) Fourier-transform infrared spectroscopy (FTIR) analysis for ACL and ACL/Fe_3_O_4_ magnetic nanocomposites before and after CV dye adsorption process; and (**b**) Vibrating sample magnetometer (VSM) analysis for Fe_3_O_4_ and ACL/Fe_3_O_4_ magnetic nanocomposites.

Fourier-transform infrared spectroscopy (FTIR) analysis for ACL and ACL/Fe_3_O_4_ magnetic nanocomposite samples was performed before and after the CV dye adsorption process and the results are shown in Figure 1a. In the structure of ACL and ACL/Fe_3_O_4_ magnetic nanocomposite, high intensity vibrations have been observed in the range of 3418–3419 cm^−1^, which is due to –OH tensile vibrations in the structure of the desired adsorbents. Also, vibrations have been observed in the range of 2852–2922 cm^−1^ which is caused by C-H vibrations in the structure of the adsorbents. In the structure of ACL, wavenumbers of 1640, 1584, 1432, 1167, 669–881, 608 and 450 cm^−1^ corresponded to vibrations of C=C or C=N, C=O, C=C or CH, C-O-C or C=O, C-H, and C-C bonds, respectively [23,24].

After placing Fe_3_O_4_ nanoparticles in the ACL structure, the range of peaks in the ACL structure was changed, which could be due to the interaction of Fe_3_O_4_ with functional groups in the ACL structure. It should also be noted that in the ACL/Fe_3_O_4_ magnetic nanocomposite structure, a high intensity peak in the range of 567 cm^−1^ has been observed due to Fe-O vibrations [25], and shows that Fe_3_O_4_ magnetic nanoparticles have been successfully incorporated into the ACL structure. After the CV dye adsorption process using ACL and ACL/Fe_3_O_4_ magnetic nanocomposite, the range and intensity of peaks in the adsorbent structure was changed, which could be due to the interaction and placement of the CV dye with the adsorbent surface. For example, after the adsorption process, the range of -OH vibrations in the ACL structure and the ACL/Fe_3_O_4_ magnetic nanocomposite changed from 3418 cm^−1^ and 3419 cm^−1^ to 3431 cm^−1^ and 3430 cm^−1^, respectively, which indicates that hydrogen bonds have been formed in the adsorption process. In addition, the results of FTIR analysis showed that functional groups such as -OH, C=O, C=C and C-O-C were effective in the CV dye adsorption process.

Magnetic property is one of the important features of magnetic adsorbents in the adsorption process, because it facilitates the separation of the adsorbent from the aqueous solution and reduces the process cost. Therefore, the magnetic properties of Fe_3_O_4_ nanoparticles and ACL/Fe_3_O_4_ magnetic nanocomposite were studied in the range of −8000 Oe to 8000 Oe and the results are shown in Figure 1b. According to the results, the magnetic saturation values for Fe_3_O_4_ nanoparticles and ACL/Fe_3_O_4_ magnetic nanocomposite were 84.3 and 32.0 emu/g, respectively.

**Figure 2:**
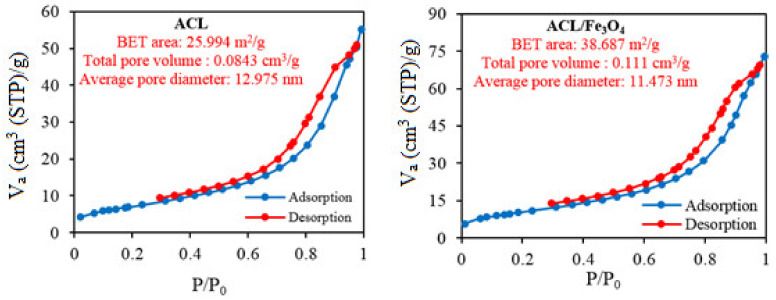
Nitrogen adsorption-desorption analysis for ACL and ACL/Fe_3_O_4_ magnetic nanocomposite samples.

Nitrogen physical adsorption experiments were studied to determine structural properties such as active surface area, pore volume and pore diameter for ACL and ACL/Fe_3_O_4_ magnetic nanocomposites and the results are shown in Figure 2. The adsorption-desorption isotherm of N_2_ for both samples followed a type IV isotherm with a residual ring at a relative pressure of P/P_0_ in the range of 0.5–1, indicating the presence of mesoporous structure of the material [26] and the adsorption of the monolayer [27]. In addition, the pore diameter size was in the range of 2–50 nm (according to the IUPAC standards) indicated that ACL and ACL/Fe_3_O_4_ magnetic nanocomposites have a mesoporous structure. Based on the analysis, the amount of specific active surface for ACL samples and ACL/Fe_3_O_4_ magnetic nanocomposites was 25.99 and 38.69 m^2^/g, respectively. The increased active level of ACL/Fe_3_O_4_ nanocomposites was due to the presence of Fe_3_O_4_ nanoparticles in the structural layers of ACL. It is also noteworthy to mention that with the placement of Fe_3_O_4_ nanoparticles, the pore volume in the ACL structure was also increased, which confirms the placement of Fe_3_O_4_ nanoparticles and the increase in the distance between the layers and the ACL pores.

The decrease in the amount of magnetic saturation in the desired magnetic nanocomposite can be due to the presence of a non-magnetic matrix (ACL) and the reduction of magnetic particles in the structure of the desired nanocomposite [28]. It is noteworthy that the absence of hysteresis ring showed that Fe_3_O_4_ nanoparticles and ACL/Fe_3_O_4_ magnetic nanocomposite have superior magnetic properties, and can be easily separated from the aqueous solution using an external magnetic field.

**Figure 3:**
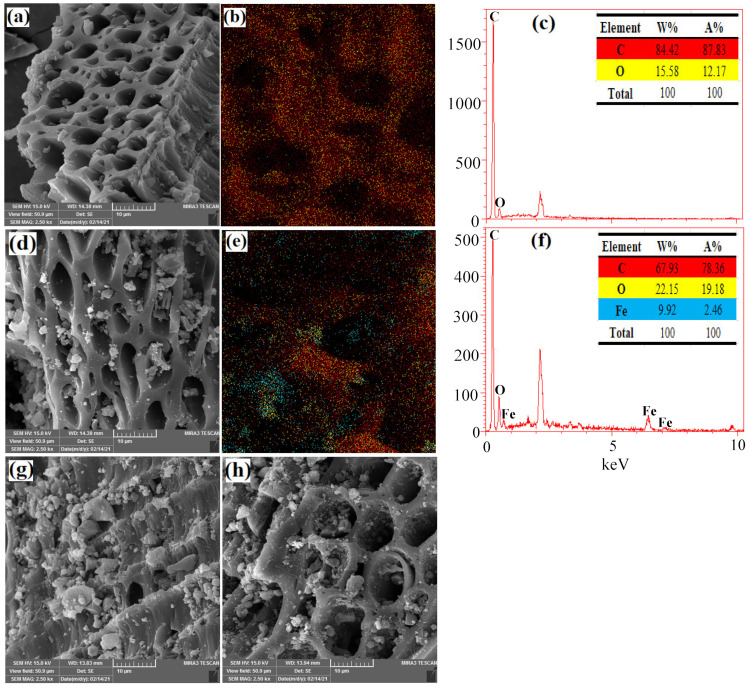
SEM, Map and EDX analyzes for: (**a**–**c**) ACL; (**d**–**f**) ACL/Fe_3_O_4_ magnetic nanocomposite; (**g**) ACL after CV dye adsorption, and (**h**) ACL/Fe_3_O_4_ magnetic nanocomposite after CV dye adsorption.

Scanning electron microscopy (SEM), Map and energy-dispersive X-ray spectroscopy (EDX) was performed to investigate the surface changes in the ACL and ACL/Fe_3_O_4_ magnetic nanocomposite before and after the CV dye adsorption process. The results Figure 3a show that ACL has a porous structure and the composition of elements C and O in its structure is 84.4% and 15.6%, respectively. Also, the results of the Map analysis showed that the elements are well distributed in the ACL structure Figure 3b. After ACL modification using Fe_3_O_4_ magnetic nanoparticles, particles of different sizes were observed in the pores and layers of the ACL, which could represent the Fe_3_O_4_ nanoparticles formed in the ACL structure Figure 3d. Also, EDX and Map analysis confirmed the presence of Fe ions (9.92%) in the ACL structure, which indicates that Fe_3_O_4_ nanoparticles have been successfully placed in the ACL structure and have a good interaction Figure 3e,f. After CV dye adsorption process using ACL and ACL/Fe_3_O_4_ magnetic nanocomposite, significant changes in the structure and pores on the adsorbent surface were observed Figure 3g,h. The changes may be due to the presence of CV dye in the adsorbent layers and pores, which confirms that ACL and ACL/Fe_3_O_4_ magnetic nanocomposite have the ability to remove CV dye from aqueous solution.

### 2.2. The Effect of pH

**Figure 4:**
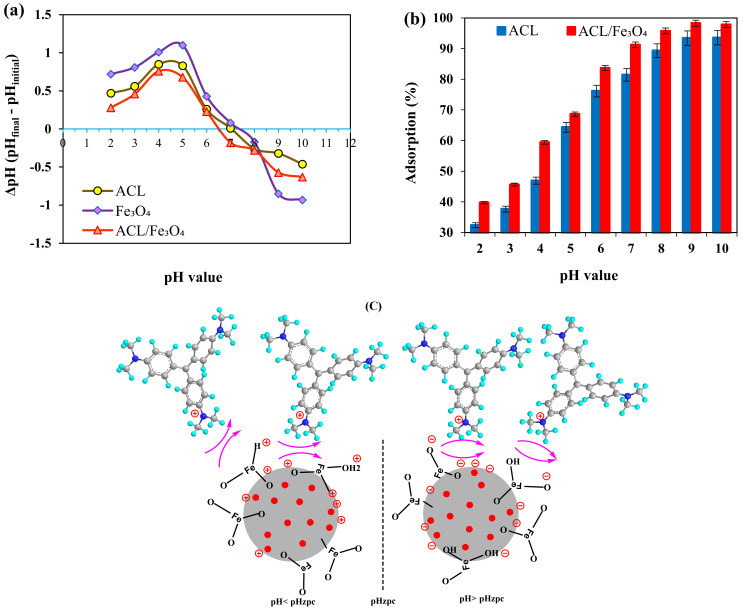
(**a**) The effect of pH on the surface loads of adsorbents; (**b**) The effect of pH on the efficiency of the adsorption process (temperature 25 °C, contact time 60 min, adsorbent dose 1.25 g/L, CV dye concentration 10 mg/L), and (**c**) the mechanism of the CV dye adsorption process in the acidic and alkaline pH ranges.

Based on previous studies, it has been reported that the initial pH is one of the effective parameters in the efficiency and adsorption capacity, because it can affect the surface loads and the degree of ionization of functional groups in the adsorbent surface [29]. For this purpose, the effect of pH on surface loads and adsorption efficiency of CV dye was investigated using both adsorbents in the initial pH range 2 to 10 Figure 4a,b.

According to the results, the pH_zpc_ values for Fe_3_O_4_, ACL and ACL/Fe_3_O_4_ magnetic nanocomposite were determined to be 7.4, 7.1 and 6.48, respectively. At pH > pH_zpc_ and pH < pH_zpc_, the adsorbent has negative and positive surface charges, respectively. According to the obtained results, by increasing the pH from 2 to 10, the CV dye adsorption efficiency using both adsorbents has increased and significant dye removal has been performed in alkaline media (pH > pH_zpc_). This condition can be due to the strong gravitational force and the reduction of repulsive force between the adsorbent surface and the CV dye [30]. It should be noted that the low efficiency of CV in acidic environments using both adsorbents can be due to the presence of H^+^ ions in aqueous solution, which competes with CV color to be located on active adsorbent sites [30,31].

The mechanism of CV dye adsorption using ACL/Fe_3_O_4_ magnetic nanocomposite in pH > pH_zpc_ and pH < pH_zpc_ values is shown in Figure 4c. The mechanism of the CV dye adsorption process in the acidic and alkaline pH ranges is shown in Figure 4c.

### 2.3. Contact Time and Kinetic Study

**Figure 5:**
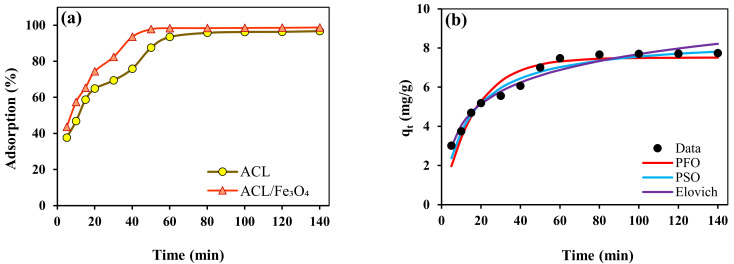

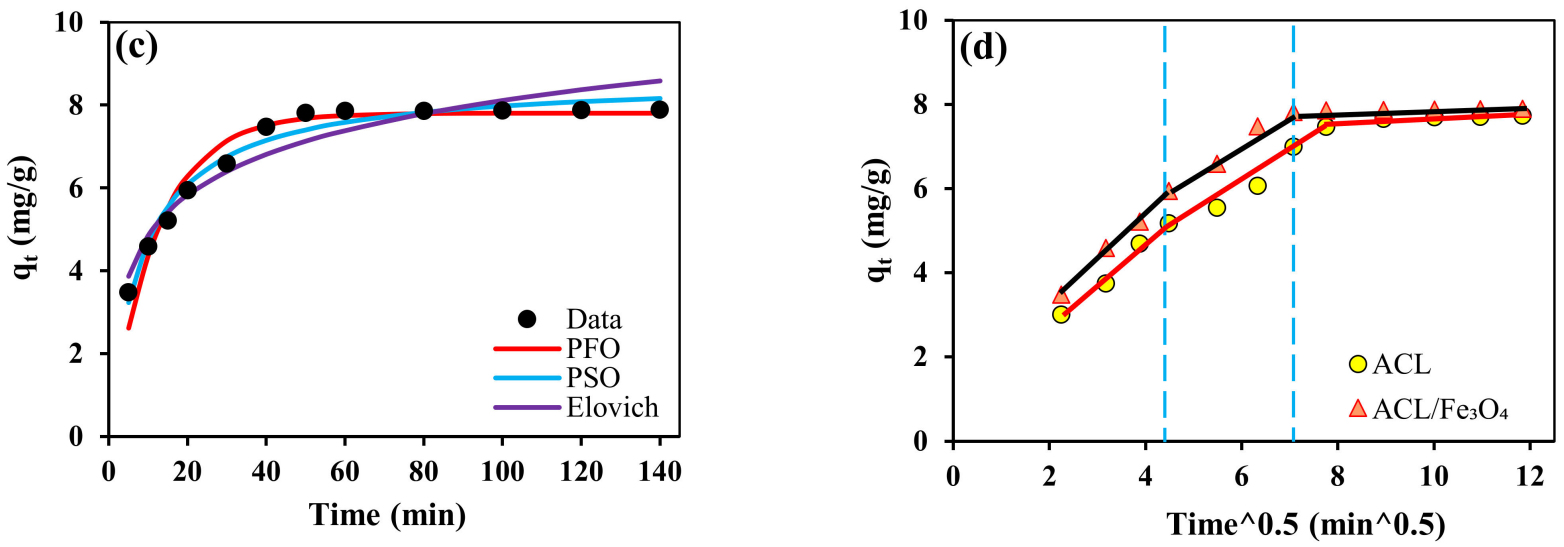
(**a**) contact time effect (pH = 9, temperature 25 °C, adsorbent dose 1.25 g/L, initial CV concentration 10 mg/L), Nonlinear relationship of PFO, PSO and intraparticle diffusion kinetic models for (**b**) ACL, and (**c**) ACL/Fe_3_O_4_ magnetic nanocomposite, and (**d**) linear relationship of Weber-Morris model for ACL and ACL/Fe_3_O_4_ magnetic nanocomposite.

One of the important and effective factors in the adsorption process is the duration time of interaction between the adsorbent surface and the contaminant, which has an effective role in the kinetics and speed of the adsorption process. The effect of contact time on CV dye adsorption efficiency using ACL adsorbents and ACL/Fe_3_O_4_ magnetic nanocomposite were investigated in the time range of 5–140 min and the results are shown in Figure 5a. According to the obtained results, the adsorption process using ACL and ACL/Fe_3_O_4_ magnetic nanocomposite has been done in three stages. The first stage (5–20 min) is faster than other stages, which can be due to the presence of empty and available active sites for the CV dye. After 80 min, the rate of adsorption process decreased significantly and the efficiency of adsorption process did not change significantly, which could indicate the equilibrium adsorption of the desired process. Therefore, the equilibrium time for the CV dye adsorption process was determined using ACL and ACL/Fe_3_O_4_ magnetic nanocomposite 80 min and 60 min, respectively. The kinetic behavior of the adsorption process can be used to find useful and effective information about the adsorption process and equilibrium time. For this purpose, to investigate the kinetic behavior of the adsorption process in the time range of 5–140 min, Pseudo-first order (PFO), Pseudo-second order (PSO), Elovich and intraparticle diffusion kinetic model (Weber-Morris) were used and their nonlinear equations are arranged as follows:

(1) $$\mathrm{Pseudo}-\mathrm{first}\;\mathrm{order}\;\left(\mathrm{PFO}\right):{\;q}_{t}=q_{e}\left(1-e^{-k_{1}t}\right)$$

(2) $$\mathrm{Pseudo}-\mathrm{second}\;\mathrm{order}\;\left(\mathrm{PSO}\right):{\;q}_{t}=\frac{k_{2}q_{e}^{2}t}{1+k_{2}q_{e}t}$$

(3) $$\mathrm{Elovich}:{\;q}_{t}=\frac{1}{\beta }\ln\left(\alpha \beta \;t\right)$$

(4) $$\mathrm{Intraparticle}\;\mathrm{diffusion}:{\;q}_{t}=K_{\mathrm{int}}\;\sqrt{t}+I$$

where, q_t_ and q_e_ are the amount of CV dye adsorption capacity per gram of dry adsorbent at any time and equilibrium time (mg/g), respectively; k_1_ is adsorption rate constant (min^−1^); k_2_ is adsorption rate constant of PSO kinetic model (g/mg/min); α is the initial adsorbance (mg/g/min); β is the desorption constant (g/mg); the K_int_ constant is the intra particle diffusion rate (mg/g min^0.5^); and the value of I is the kinetic constant of the intra particle diffusion kinetic model that gives ideas about the boundary layer thickness.

The linear and nonlinear relationship of kinetic models for the CV adsorption process using ACL and ACL/Fe_3_O_4_ magnetic nanocomposite were shown in Figure 5b,c and the kinetic variables specified are listed in Table 1. The results of the kinetic behavior study showed that the PSO model has a greater ability to describe the kinetic behavior of the process than other kinetic models, because it has a higher correlation coefficient (R^2^) and higher adsorption capacity (q_e.cal_), and the root-mean-square error (RMSE) is less. In addition, the α parameter for the CV dye adsorption process using the ACL/Fe_3_O_4_ magnetic nanocomposite is higher than that of ACL, which shows that ACL/Fe_3_O_4_ magnetic nanocomposite has a greater tendency to interaction CV dye and is also consistent with experimental results.

In addition, the correlation coefficient (R^2^) values determined using the Alovitch model for ACL and ACL/Fe_3_O_4_ magnetic nanocomposite was 0.9642 and 0.9156, respectively, which indicates that chemical mechanisms and ion exchange are also effective in the adsorption process [32], and by modifying ACL using Fe_3_O_4_ nanoparticles the effect of chemical mechanism and ion exchange is reduced. In the adsorption process, PFO and PSO models are not able to investigate and determine the mechanism of the adsorption process. There are several stages involved in the adsorption process, and one of the important steps that can control the speed of the adsorption process is film diffusion and intraparticle diffusion [33].

For this purpose, the intraparticle diffusion model is used to explain the different stages of the adsorption process. The line diagram of the intra-particle diffusion model for the CV dye adsorption process using the adsorbents used in Figure 5d is shown. When the line passes through the origin, it can be suggested that intraparticle diffusion is the only mechanism controlling the adsorption process. The linear relationship of the Weber-Morris model shows that the adsorption process has three stages that are consistent with the experimental results. Also, the results showed that intraparticle diffusion is not the only mechanism controlling the adsorption process and the boundary layer is a mechanism control in a multi-stage system [34]. The first stage (2.236 ≤ t^0.5^ ≤ 5.22) is related to the film diffusion mechanism that the contaminant immediately enters the adsorbent surface [35]. The second (5.186 ≤ t^0.5^ ≤ 7.824) and the third stage (7.824 ≤ t^0.5^ ≤ 7.9908) represent intraparticle diffusion and equilibrium condition, respectively [36].

### 2.4. Effect of Initial CV Content and Isotherm Study

**Figure 6:**
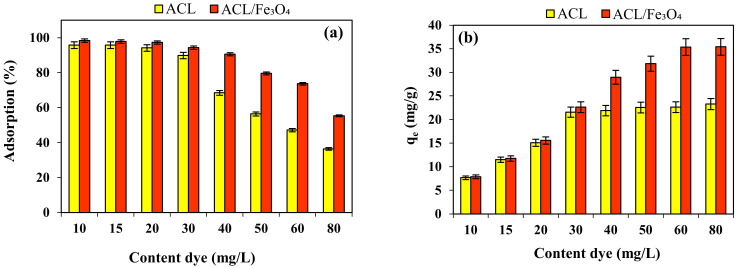

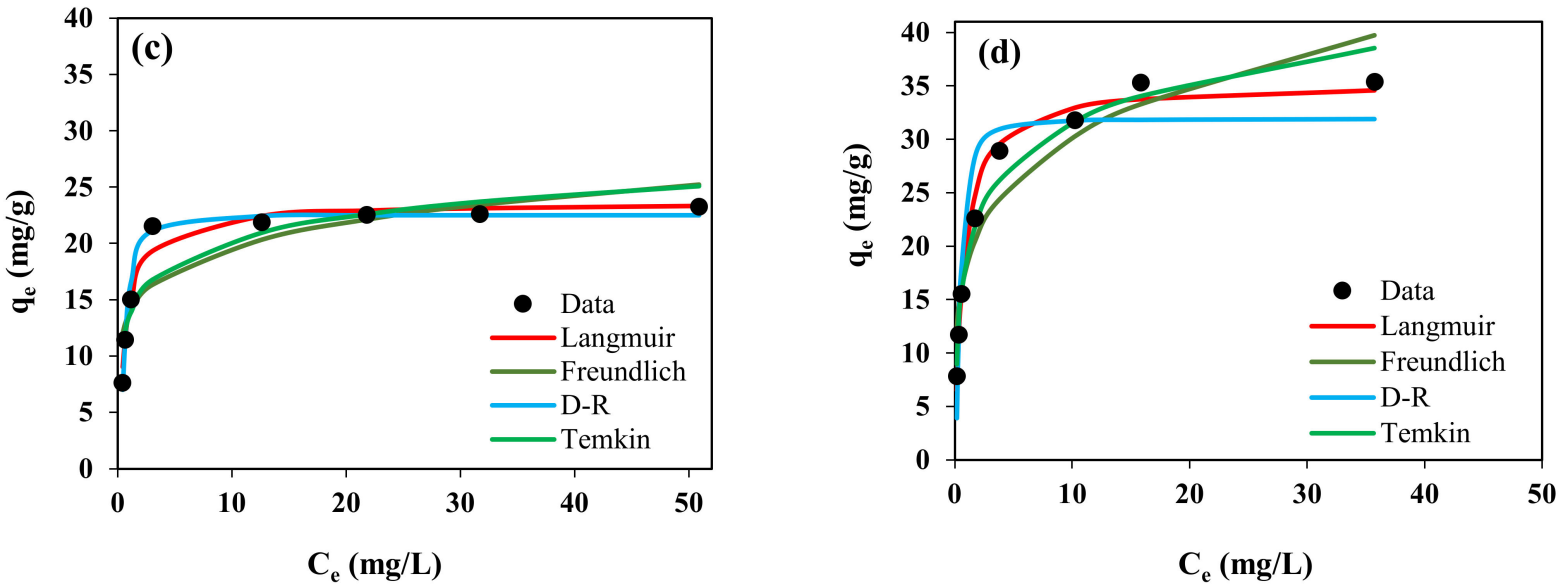
(**a**,**b**) Effect of initial CV concentration on efficiency and adsorption capacity (pH = 9, contact time for ACL and ACL/Fe_3_O_4_ magnetic nanocomposite 80 and 60 min, temperature 25 °C, adsorbent dose 1.25 g/L), Nonlinear relationship of isotherm models for CV dye adsorption process using (**c**) ACL and (**d**) ACL/Fe_3_O_4_ magnetic nanocomposite. Data are mean of triplicate measurements. (**a**,**b**) the error bars indicate SD values.

The initial concentration of the dye can have a significant effect on the efficiency and adsorption capacity of the process due to providing the necessary driving force for mass transfer between the aqueous phase (aqueous solution containing dye) and the solid phase (adsorbent). Figure 6a,b shows the effect of the initial concentration of CV dye in the range of 10–80 mg/L on the efficiency and adsorption capacity. The results showed that with increasing the initial concentration of CV dye, the efficiency of adsorption process using for ACL and ACL/Fe_3_O_4_ magnetic nanocomposite decreased from 95.78% and 98.34% to 36.38% and 55.34%, respectively. The high removal efficiency at low concentrations of dye solution can be due to the greater interaction of dye molecules with the surface and active sites in the adsorbent structure. In addition, the decrease in adsorption efficiency at high concentrations of CV dye could be due to the saturation of adsorbent active sites at high concentrations of CV, limited active adsorbent sites or increased repulsive electrostatic force between the adsorbent surface and CV dye in aqueous solution [37]. Although with increasing the initial concentration of CV dye, the efficiency of the adsorption process has decreased, but the adsorption capacity of adsorbents has increased. Increasing the adsorption capacity of adsorbents by increasing the initial concentration of CV dye solution can be due to the increased slope of the driving force of mass transfer from aqueous solution to the surface of adsorbents, which has increased the penetration of dye molecules in the internal pores and active sites in the structure of adsorbents [38]. Different isotherm models can be used to investigate the equilibrium behavior between the desired pollutant and the adsorbents used, as well as to determine the type of process.

In this study, the Langmuir, Freundlich, Temkin and Dubinin–Radushkevich (D-R) isotherm models were used to investigate the equilibrium behavior of the CV adsorption process using ACL and ACL/Fe_3_O_4_ magnetic nanocomposite. The Langmuir isotherm model is based on the assumption that monolayer adsorption occurs at homogeneous active sites in the adsorbent structure; while the Freundlich model is based on the assumption that adsorption occurs at heterogeneous and non-uniform surfaces. The D-R model assumes that the adsorbent surface used is heterogeneous and is used to show the chemical and physical adsorption mechanism of the process [39]. The nonlinear equations of the isotherm models used in this study are as follows:

(5) $${\mathrm{Langmuir}:\;q}_{e}=\frac{q_{m}k_{l}C_{e}}{1+k_{l}C_{e}},{\;R}_{L}=\frac{1}{1+K_{L}+C_{o}}$$

(6) Freundlich : qe=kfCe1n

(7) $$D-{R:q}_{e}=q_{m}\exp(-{\beta \varepsilon }^{2}),\;\varepsilon =\mathrm{RTln}(1+\frac{1}{C_{e}})$$

(8) $${\mathrm{Temkin}:q}_{e}=\mathrm{Bln}(A\times C_{e}),\;B=\frac{\mathrm{RT}}{b_{T}}$$

where, q_e_ is the equilibrium adsorption capacity (mg/g); q_m_ is the maximum adsorption capacity of the monolayer (mg/g); k_L_ is the Langmuir adsorption constant, which represents the binding energy (L/mg); k_F_ and n are Freundlich model constants; b_T_ (kJ/mol) and A_T_ (l/g) are Obedience model constants, R is universal constant of gases, T (K) is absolute temperature, ε is polanyi coefficient, β is activity coefficient (mol^2^/J^2^), which represents the free energy of adsorption.

The nonlinear relationship of the isotherm models used, the constants and the determined variables are shown in Figure 6c,d and Table 2, respectively.

As the results show, in the CV dye adsorption process using ACL and ACL/Fe_3_O_4_ magnetic nanocomposite, D–R and Langmuir isotherm models have more ability to describe equilibrium data, respectively, which show that heterogeneous and homogeneous surfaces are effective in the adsorption process, respectively. The values of n and R_L_ parameters determined using the Freundlich and Langmuir isotherm models showed that the adsorption process using both types of adsorbents used is desirable and physical. The K_f_ parameter for the CV adsorption process was determined using ACL and ACL/Fe_3_O_4_ magnetic nanocomposite to be 13.78 mg/g (L/mg)^1/n^ and 18.25 mg/g (L/mg)^1/n^, respectively, which shows that the bond between the cationic dye of CV and the surface of the ACL/Fe_3_O_4_ magnetic nanocomposite is greater and stronger compared to ACL [40]. Also, the average energy value (E) was determined using ACL and ACL/Fe_3_O_4_ magnetic nanocomposite 1.972 kJ/mol and 2.364 kJ/mol, respectively, which shows that the adsorption process is physical (E < 8 kJ/mol) [29,41,42] and is well consistent with the results of the ΔG parameter. The determined parameters (b_T_ and A) using Temkin model showed that the interactions between the adsorbent surface and the desired cationic dyes are weak and may be a physical adsorption process.

### 2.5. Effect of Absorbent Dose

**Figure 7:**
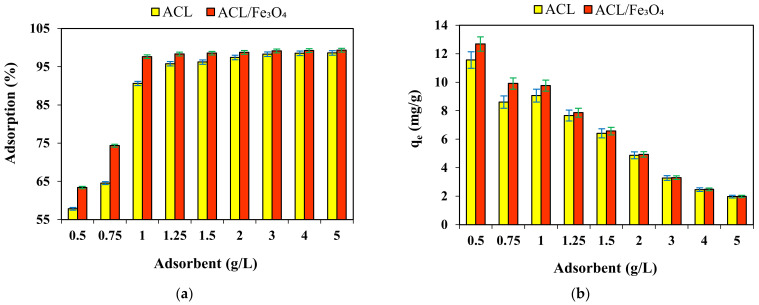
Effect of adsorbent dose on (**a**) adsorption efficiency, and (**b**) adsorption capacity (pH = 9, temperature 25 °C, initial CV dye concentration 10 mg /L, contact time for ACL and ACL/Fe_3_O_4_ magnetic nanocomposite 80 and 60 min, respectively). Data are mean of triplicate measurements and error bars indicate SD values.

Absorbent dose is another effective parameter in the adsorption process that can affect the efficiency and adsorption capacity of the adsorbent. The effect of adsorbent dose on CV dye efficiency and adsorption capacity was investigated using ACL and ACL/Fe_3_O_4_ magnetic nanocomposite in the range of 0.5–5 g/L and the results are shown in Figure 7. The results showed that with increasing the adsorbent dose of ACL and ACL/Fe_3_O_4_ magnetic nanocomposite from 0.5 g/L to 5 g/L, the adsorption efficiency increased from 57.82% and 63.41% to 98.61% and 99.34%, respectively. Increasing the adsorption efficiency by increasing the adsorbent dose can be due to increasing the number of unsaturated and available active sites [43,44]. According to Figure 7a, the optimal adsorbent dose for ACL and ACL/Fe_3_O_4_ magnetic nanocomposite was determined to be 2 g/L and 1.25 g/L, respectively. After the optimal dose of adsorbent, the efficiency of the adsorption process did not change significantly, which can be caused by various factors such as decreasing the concentration of CV dye in aqueous solution and non-contact of dye with active adsorbent sites, adsorbent particles collide with each other and reduce the active surface [43]. In addition, according to the results Figure 7b, it can be mentioned that with increasing the adsorbent dose, the adsorption capacity of adsorbents used in the CV dye adsorption process has decreased, which can be due to the vacancy of active sites of adsorbents in high doses, because the concentration of pollutants is constant, but the number of active sites has increased and not all active sites of adsorbents have been used well [45].

### 2.6. Effect of Temperature and Thermodynamic Study

**Figure 8:**
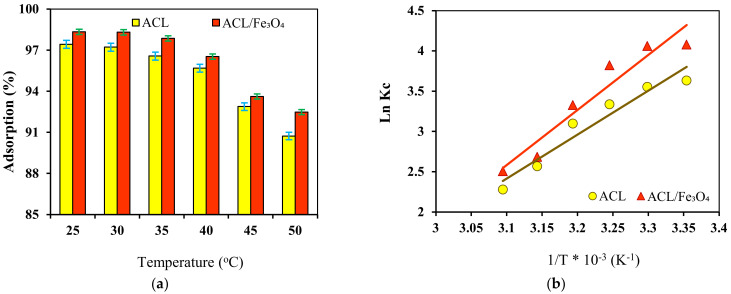
(**a**) The effect of temperature on the adsorption efficiency (pH = 9, contact time for ACL and ACL/Fe_3_O_4_ magnetic nanocomposite 80 and 60 min, respectively, adsorbent dose for ACL and ACL/Fe_3_O_4_ magnetic nanocomposite 2 and 1.25 g/L, respectively, initial dye concentration of 10 mg/L); (**b**) the linear relation of van’t Hoff equation to determine thermodynamic parameters. Data are mean of triplicate measurements. (**a**) error bars indicate SD values.

Temperature is one of the important and effective parameters in the adsorption process and it has been shown that this parameter affects the transfer process and the adsorption kinetics of dyes. The effect of temperature on CV dye adsorption efficiency was investigated using ACL and ACL/Fe_3_O_4_ magnetic nanocomposite in the range of 25–50 °C Figure 8a. The results showed that with increasing the temperature from 25 °C to 50 °C, the efficiency of CV dye adsorption process using ACL and ACL/Fe_3_O_4_ magnetic nanocomposite decreased from 97.43% and 98.34% to 90.73% and 92.48%, respectively. This indicates the exothermic nature of the CV dye adsorption process using the adsorbents. Decreasing the efficiency of the adsorption process with increasing temperature can be due to the weakening of the physical bonds between the dye molecule and the active sites of the adsorbent, increase the solubility of CV dye in aqueous solution, which makes the interactions between the dye molecule and the solvent stronger than the adsorbent [46]. Therefore, a temperature of 25 °C was determined as the optimal temperature for the CV dye adsorption process using ACL and ACL/Fe_3_O_4_ magnetic nanocomposite. Van’t Hoff equation was used to investigate the behavior and determine thermodynamic parameters such as enthalpy (ΔH), entropy (ΔS) and Gibbs free energy (ΔG) in the temperature range of 25–50 °C Figure 8b and the results are shown in Table 3.

According to the results, the value of ΔG for the CV dye adsorption process using ACL and ACL/Fe_3_O_4_ magnetic nanocomposite has a negative value, which shows that the adsorption process is spontaneous and possible [47]. Also, the value of the parameter ΔG for the adsorption process is in the range of kJ/mol −20 < ΔG < 0, which shows that the adsorption process using both types of adsorbents is physical [48]. Also, the value of ΔH parameter for CV dye adsorption process was determined using ACL and ACL/Fe_3_O_4_ magnetic nanocomposite −45.382 kJ/mol and −56.901 kJ/mol, respectively, which indicates that the adsorption process is exothermic. Also, the value of the ΔS parameter was negatively determined for the adsorption process, which indicates that random collisions and irregularities at the adsorbent surface are reduced during the adsorption process. According to the results, it seems that the parameter ΔH has a greater effect than the ΔS in determining the negative value of ΔG [49].

### 2.7. Comparison of Adsorption Capacity

The adsorption capacity of different adsorbents used in the adsorption process, depending on the primary source of the adsorbent, the process conditions of adsorption, adsorption modification, and the type of contaminant can be different. Today, extensive research has been done to find adsorbents with good adsorption capacity and low economic cost. Due to the mentioned reasons, in the present study, the adsorption capacity of ACL/Fe_3_O_4_ and ACL magnetic nanocomposites was compared with other natural and synthetic adsorbents used in the CV dye adsorption process and the results are reported in Table 4. The results show that the ACL/Fe_3_O_4_ and ACL magnetic nanocomposite has a higher ability to absorb CV from aqueous solutions compared to many natural and synthetic adsorbents and can be used as an effective adsorbent in the CV dye adsorption process.

## 3. Materials and Methods

### 3.1. Materials

Lemon wood was obtained from a local garden and used as a primary source in the production of activated carbon. Sodium hydroxide (NaOH), hydrochloric acid (HCl), iron (III) chloride hexahydrate (FeCl_3_·6H_2_O), iron (II) tetrachloride (FeCl_2_·4H_2_O), crystal violet (CV) dye was prepared from Merck (Darmstadt, Germany). To prepare a standard CV dye solution, a certain amount of dye was dissolved in deionized water. To prepare aqueous solutions containing CV dye for adsorption test, stock solution was diluted with deionized water and used. Deionized water was used for solutions in all stages of the experiments.

### 3.2. Synthesis of Adsorbents

**Figure 9:**
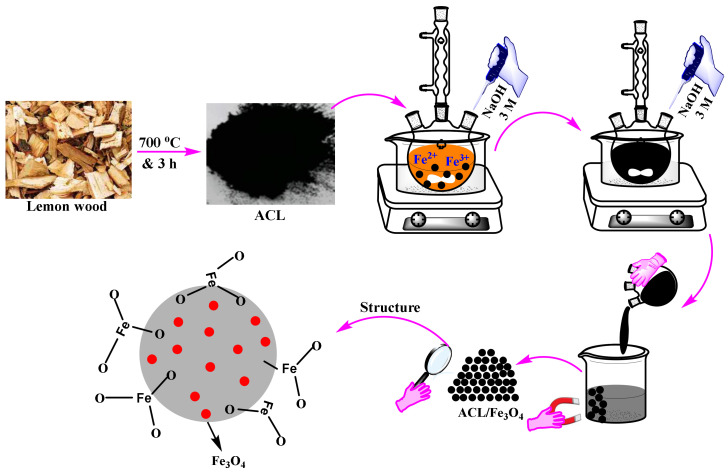
A schematic illustration of the productions process and structure of ACL/Fe_3_O_4_ magnetic nanocomposite.

The lemon wood was washed with tap water and dried at 105 °C for 48 h. The dried wood was burnt at 700 °C for 3 h to prepare the desired carbon. The prepared carbon was then pulverized using a kitchen mill and stored at room temperature. In order to produce ACL/Fe_3_O_4_ magnetic nanocomposite, co-precipitation method was used. For this purpose, at first, 2 g of ACL was added to an aqueous solution (100 mL) containing Fe (III) and Fe (II) ions in a 1:2 molar ratio and stirred for 40 min using a magnetic stirrer. At the end of this time, NaOH solution with a concentration of 3 M (30 mL) was added dropwise and stirred for 50 min at 80–90 °C using a magnetic stirrer. The produced magnetic nanocomposite was isolated using an external magnetic field and collected after washing for several times followed by drying at 105 °C for 24 h. The obtained powder of magnetic nanocompositewas stored at ambient temperature. Figure 9 shows an overview of the ACL/Fe_3_O_4_ magnetic nanocomposite synthesis process.

### 3.3. Characteristics of Adsorbents

Fourier-transform infrared spectroscopy (FTIR) analysis using a Tensor 27, spectrophotometer (Bruker, Bremen, Germany) [67] was used to characterize the chemical structure of ACL and ACL/Fe_3_O_4_ magnetic nanocomposite in the range of 4000–400 cm^−1^. Nitrogen physical adsorption experiments (BET test) using Accelerated Surface Area and Porosity (ASAP) measurement (model ASAP 2020, Micrometrics Instruments, Norcross, GA, USA) was used to determine the structural properties of ACL samples and ACL/Fe_3_O_4_ magnetic nanocomposites such as active surface area, pore volume and pore diameter. The morphology and porous structure of ACL and ACL/Fe_3_O_4_ nanoparticles were investigated using scanning electron microscopy (MIRA3, TESCAN, Brno, Czech Republic) with energy dispersive X-ray spectroscopy (EDX). Vibrating sample magnetometer (VSM) analysis (model 7400-S, Lake-Shore Cryotronics, Westerville, OH, USA) was performed to evaluate the magnetic properties of Fe_3_O_4_ and the ACL/Fe_3_O_4_ magnetic nanocomposite in the range of −8000 Oe to 8000 Oe.

### 3.4. Adsorption Study

CV dye adsorption was investigated using ACL adsorbents and ACL/Fe_3_O_4_ magnetic nanocomposite in 100 mL tubes and discontinuously. In the process of adsorption of CV dye, the effect of various parameters such as pH (2–10), contact time (5–140 min), temperature (25–50 °C), adsorbent dose (0.5–5 g/L) and initial concentration of CV dye (10–80 mg/L) were evaluated on the efficiency of the adsorption process. To investigate the behavior of the adsorption process in the desired pH range, a certain amount of ACL and ACL/Fe_3_O_4_ magnetic nanocomposite was added to 50 mL of aqueous solution containing CV dye at the desired pH and stirred for 60 min. At the end of the time, the adsorbents were separated from the aqueous phase and the amount of CV dye remaining in the aqueous medium was determined using a UV-vis spectrophotometer (model Cary 100, Agilent Technologies, Santa Clara, CA, USA) at 592 nm and the maximum adsorption efficiency was determined as the optimal value. After determining the optimal pH, other parameters were examined at the optimal pH and in the desired ranges, and the maximum efficiency was determined. At each stage, the efficiency and adsorption capacity were determined from Equations (9) and (10), respectively:

(9) $$R\left(\%\right)=\left(\frac{{\mathrm{CV}}_{i}-{\mathrm{CV}}_{e}}{{\mathrm{CV}}_{i}}\right)\times 100$$

(10) $$q_{e}=\left({\mathrm{CV}}_{i}-{\mathrm{CV}}_{e}\right)\times \frac{V}{m}$$

where, CV_i_ and CV_e_ are the initial concentration and final concentration of CV dye (mg/L), V is the volume of solution used (L) and m (g/L) is the amount of adsorbent used, and q_e_ (mg/L) is the adsorption capacity of the adsorbents.

## 4. Conclusions

In this study, activated carbon prepared from lemon wood (ACL) and magnetic nanocomposite of ACL were used as an effective and low-cost adsorbent in the adsorption process of cationic crystal violet (CV) dye from aqueous solution. The results of FTIR analysis showed functional groups such as C=O, C-O, Fe-O and -OH in the structure of ACL and ACL/Fe_3_O_4_ magnetic nanocomposites, which can be effectively used in the process of CV dye adsorption. VSM analysis also showed superior magnetic properties of Fe_3_O_4_ nanoparticles and ACL/Fe_3_O_4_ magnetic nanocomposite that can be easily separated from the aqueous solution using an external magnetic field. The results of different equilibrium models showed that the CV dye adsorption equilibrium data using ACL and ACL/Fe_3_O_4_ magnetic nanocomposite followed the D-R and Langmuir models, respectively. Based on isotherm studies, it was shown thatmonolayer and homogeneous surfaces are more effective than heterogeneous surfaces. ACL/Fe_3_O_4_ magnetic nanocomposite showed significantly higher adsorption capacity than ACL nanoparticles. In addition, parameters such as n, R_L_, E and b_T_ showed that the adsorption process is desirable and physical and the CV dye can be easily separated from the surface of the desired adsorbents. The kinetic data of the adsorption process for both types of adsorbents followed the PSO kinetic model, which shows that chemical reactions can also be effective in the adsorption process. The study of thermodynamic parameters was in good agreement with the experimental data and showed that the CV dye adsorption process is spontaneous, exothermic, and during the adsorption process the amount of irregularity is reduced. Concluding, ACL and ACL/Fe_3_O_4_ magnetic nanocomposite can be introduced and used as an effective and efficient adsorbent in removing CV dye from aqueous solution and industrial wastewaters.

## Figures and Tables

**Table 1:** Kinetics constants and parameters determined using kinetic models for the adsorption process.

Kinetic Model	Adsorbent	
	ACL	ACL/Fe_3_O_4_
Pseudo-first order		
q_e cal_	7.51	7.808
K_P1*s*t_	0.0608	0.0819
R^2^	0.9151	0.9484
RMSE	0.5142	0.3687
Pseudo-second order		
q_e.cal_	8.545	8.648
K_P2s*t*_	0.009	0.0138
R^2^	0.9664	0.9730
RMSE	0.3235	0.2666
Elovich equation		
α (mg/g min)	2.049	4.34
β (g/mg)	0.6328	0.7057
R^2^	0.9642	0.9156
RMSE	0.3337	0.4719
Intraparticle diffusion		
K_i,1_ (mg/g min^1/2^)	1.0021	1.0836
I_1_ (mg/g)	0.7179	1.0916
R^2^	0.9887	0.9965
K_i,2_ (mg/g min^1/2^)	0.8855	0.7777
I_2_ (mg/g)	0.6332	2.4091
R^2^	0.9807	0.954
K_i,3_ (mg/g min^1/2^)	0.0247	0.0081
I_3_ (mg/g)	7.4444	7.8024
R^2^	0.9495	0.9696

**Table 2:** Constant isotherms and parameters determined for the CV dye adsorption process.

Models		Adsorbent	
	Parameters	ACL	ACL/Fe_3_O_4_
Langmuir	q_m_ (mg/g)	23.64	35.31
	K_L_ (L/mg)	1.469	1.366
	R_L_	0.008–0.063	0.009–0.68
	R^2^	0.9704	0.9826
	RMSE	1.122	1.435
Freundlich	n	6.5	4.595
	K_f_ (mg/g (L/mg)^1/n^)	13.78	18.25
	R^2^	0.78	0.891
Dubinin–Radushkevich (D–R)	RMSE	3.06	3.591
	E (kJ/mol)	1.972	2.364
	q_m_ (mg/g)	22.53	31.91
	β (mol^2^/J^2^)	1.285 × 10^−7^	8.969 × 10^−8^
	R^2^	0.9838	0.8828
Temkin	RMSE	0.8293	3.723
	b_T_ (kJ/mol)	0.837	0.45
	A_T_ (L/g)	94.77	30.79
	R^2^	0.8415	0.9737
	RMSE	2.597	1.904

**Table 3:** Thermodynamic parameters for the CV dye adsorption process.

Adsorbent	T (°C)	ΔG° (KJ/mol)	ΔH° (KJ/mol)	ΔS° (J/mol·K)
ACL	25	−9.011	−45.382	−120.594
	30	−8.958		
	35	−8.551		
	40	−8.071		
	45	−6.793		
	50	-6.128		
ACL/Fe_3_O_4_	25	−10.117	−56.901	−154.915
	30	−10.241		
	35	−9.793		
	40	−8.666		
	45	−7.105		
	50	−6.742		

**Table 4:** Comparison of ACL adsorption capacity and ACL/Fe_3_O_4_ magnetic nanocomposite with other adsorbents used in CV removal process.

Adsorbent	q_e_ (mg/g) CV Dye	Reference
Magnetite alginate	37.5	[50]
P(AAm-MA)/MMT	20.36	[51]
Starch-g-poly (acrylic acid)/ZnSe	10	[52]
Poly (acrylamide)-kaolin composite hydrogel	23.8	[2]
Polyvinyl alcohol/agar/maltodextrin	19.17	[53]
Guar gum/bentonite bionanocomposite	167.929	[54]
Soil-silver nanocomposite	1.918	[55]
Activated carbon	35.64	[56]
NaOH-modified rice husk	44.876	[57]
Leaf biomass of *Calotropis procera*	4.14	[58]
TLAC/Chitosan composite	0.269–2.375	[59]
Chitin nanowhiskers	59.52	[60]
AC-Fe_2_O_3_·NPLs	16.5	[61]
Chitin-psyllium based aerogel	227.11	[62]
Poly(benzofuran-co-arylacetic acid)-FA	25.10	[63]
Azolla and fig leaves modified with magnetite iron oxide nanoparticles	25	[64]
Solid waste of rosewater extraction	78.24	[65]
*Eucalyptus camdulensis* sawdust-derived biochar (*Ec*-bio)	54.7	[66]
ACL	23.64	This study
ACL/Fe_3_O_4_ magnetic nanocomposite	35.31	This study

## Data Availability

The data presented in this study are available on request from the corresponding authors.

## Sample Availability

Samples of the compounds ACL and ACL/Fe_3_O_4_ are available from the authors.
